# Plant food allergy – is it time to see the seeds?

**DOI:** 10.1186/2045-7022-5-S3-P26

**Published:** 2015-03-30

**Authors:** Sigrid Sjölander, Anna Rylander, Otti Bengtsson-Gref, Peter Brostedt, Hillevi Englund

**Affiliations:** 1 Thermo Fisher Scientific, R&D, Uppsala, Sweden

## 

Allergic reactions to fruits and vegetables are frequently observed, however skin prick tests and serological analyses may not always confirm allergy. Recent advances have identified the seeds as potential allergen sources. Seeds are generally rich in storage proteins like 7S/11S globulins and 2S albumins, which are allergens known to be linked to severe reactions. For a number of plant food, seeds are ingested, both intentionally and accidentally. In addition, seeds content can potentially leak during food processing leading to unintentional storage protein contamination.

In this study, three unrelated seed rich species were chosen: strawberry, tomato and bell pepper. For tomato storage proteins have been described, but not for strawberry and bell pepper. Here, fruit pulp and seed were separated, extracted and analyzed. Analytical gel filtration revealed different protein patterns in pulp and seeds for all three species, confirmed also by SDS-PAGE analysis.

IgE-analyses towards pulp and seed extract respectively with sera from patients with documented allergy were performed. Differences in IgE-binding to seeds and pulp extracts were demonstrated for all patient sera.

Given the near 20 nuts, legumes, seeds and grains with IUIS-registered storage proteins, it seems likely that there are storage proteins present also in seeds from other fruits and vegetables. Here, the protein patterns for pulp and seed differed in the biochemical analyses. Further, clear differences in IgE-binding towards seed and pulp were observed in sera from 20 allergic patients, encouraging further studies revealing the clinical importance of seeds in plant food allergy.

